# Neuroprotective Effect of *Cyperi rhizome* against Corticosterone-Induced PC12 Cell Injury via Suppression of Ca^2+^ Overloading

**DOI:** 10.3390/metabo9110244

**Published:** 2019-10-23

**Authors:** Hongmei Jia, Yang Liu, Meng Yu, Hai Shang, Hongwu Zhang, Liyan Ma, Tao Zhang, Zhongmei Zou

**Affiliations:** Institute of Medicinal Plant Development, Chinese Academy of Medical Sciences and Peking Union Medical College, Beijing 100193, China; jia090909@126.com (H.J.); 15002258334@163.com (Y.L.); myu@implad.ac.cn (M.Y.); hshang@implad.ac.cn (H.S.); hwzhang@implad.ac.cn (H.Z.); lyma@implad.ac.cn (L.M.); tzhang@implad.ac.cn (T.Z.)

**Keywords:** *Cyperi rhizome*, cell metabolomics, corticosterone-induced PC12 cells, Ca^2+^ overloading

## Abstract

Cyperi Rhizoma (CR) is a well-known functional food and traditional herbal medicine in Asian countries for the treatment of menstrual or emotional disturbances in women. Recent studies have shown the pharmacological effects of CR on neuronal diseases, such as Parkinson’s disease (PD) and depression. Thus, the neuroprotective effect of CR might play a vital role in exerting its effect. Here, corticosterone-induced PC12 cells were applied to screen the active fraction of CR and evaluate its neuroprotective effect. The results indicated that the fraction containing medium-polarity chemical constituents (CR-50E) displayed the best protection effect. CR-50E could increase the cell viability and reduce cell apoptosis through inhibiting oxidative stress and decreasing the lactate dehydrogenase LDH release induced by corticosterone. Further, the mechanism of action was explored by cell metabolomics. The result showed CR-50E mediated the sphingolipids metabolism of corticosterone-induced PC12 cells, which suggested inhibition of Ca^2+^ overloading may involve the protection of CR-50E against cell damage. The expression levels of three key proteins in calcium transport, including phospholipase A2 (PLA2), calcium/calmodulin independent protein kinase II (CaMK II), and caspase-3, confirmed the above result by Western blot. The findings suggest that CR-50E can suppress the disequilibrium of calcium homeostasis-mediated apoptosis by improving the abnormal sphingolipids metabolism as well as remedying the damage of the cell membrane.

## 1. Introduction

Cyperi rhizoma (CR), the rhizome of *Cyperus rotundus* L., is extensively used as a food flavoring agent in curries, pickles, and several bakery products in Asian countries [1]. It is also commonly used as a traditional herbal medicine for menstrual or emotional disturbances in women and stomach disorders [2]. Usually, it has been prescribed as a crucial herb in traditional Chinese medicine (TCM) formulas for the treatment of gynecology or neurological disorders, such as Xiang-Fu-Si-Wu Decoction, Chaihu-Shu-Gan-San, Yueju Pill, and Xiang-Su-San [3,4,5,6]. Several studies have shown anti-inflammatory, antibacterial, and antiapoptotic [2,7] effects of CR. Recent research found that its water extract attenuated the neuronal damage of 6-hydroxydopamine-induced primary dopaminergic cell injury [8], and it also inhibited the reduction in nigrostriatal dopamine genic neurons in estrogen-deprived mice treated with 1-methyl-4-phenyl-1, 2, 3, 6-tetrahydropyridine [9]. Meanwhile, the 95% ethanol extract, ethyl acetate, and n-butanol fractions from the 70% ethanol extract of CR were reported to have an antidepressant effect through significantly shortening immobility time in the swimming test and the tail suspension test in mice [10,11]. Additionally, the volatile oil site of CR displays an improvement in the anxiety behaviors of mice exposed to chronic restraint stress [12]. However, it is unclear which constituents, lower or higher polarity, are responsible for those effects of CR.

The damage of the hippocampus neuron is the key part in plasticity regulation of synapses and plays a critical role in the mechanism of neuropsychiatric disorders [13]. Glucocorticoids (GCs) are important risk factors for neurological disorders. The existing research results showed that the elevated circulating glucocorticoid induced by chronic overstress was closely related to the damage of hippocampal dentate gyrus neurons. [14]. The rat pheochromocytoma cell (PC12), which contains GC receptors and mRNA, is similar in morphology and function to sympathetic neurons. Corticosterone-induced cytotoxicity of differentiated rat PC12 cells is a classical injury model which was applied to simulate the hippocampal neuron damage state induced by GC. [15,16]. Metabonomics can prove the dynamic metabolic response to environmental stimuli or genetic modification [13]. Cell or tissue has a unique metabolic profiling that can illuminate specific information about the organ or tissue [17]. The cell metabolism is a huge network of chemical reactions, and a finely coordinated network of biochemical reactions. Cell metabonomics can reveal information which occurs within cells, such as changes in metabolic pathways, biochemical reactions, and processes [18]. Therefore, cell metabonomics has become an important tool for studying cell responses to explore potential mechanisms of drug action on metabolic pathways, which can be used for discovery of drug targets and investigation of drug effects [19,20,21].

In this study, we applied corticosterone-induced PC12 cells to screen the active fraction of CR and evaluate its cellular protective effect. Cell metabolic profiling and the expression levels of key proteins in major pathways were investigated to explore the mechanism of CR by cell metabolomics and Western blot.

## 2. Materials and Methods

### 2.1. Chemicals and Reagents

3-(4, 5-dimethylthiazol-2-yl)-2, 5-diphenyltetrazolium bromide (MTT) and corticosterone were purchased from Sigma (St. Louis, MO, USA). Dulbecco’s Modified Eagle culture medium (DMEM), fetal bovine serum (FBS), heat-inactivated horse serum, penicillin, and streptomycin were purchased from Gibco (Grand Island, NY, USA). Lactate dehydrogenase (LDH) assay kit was purchased from Nanjing Jiancheng Bioengineering Institute (Nanjing, China). Antibodies against Phospholipase A2 IIA (PLA2), calcium/calmodulin-dependent protein kinase II (CaMK II), pro-caspase-3, BDNF, and GAPDH were supplied by Epitomics (Epitomics Inc., Burlingame, USA). High Performance Liquid Chromatography (HPLC) grade dimethyl sulfoxide (DMSO), acetonitrile, and methanol were purchased from Fisher (New Jersey, USA). HPLC-grade formic acid was purchased from Tedia (Fairield, USA). Ultrapure water (18.2 MΩ) was prepared with a Milli-Q water purification system (Millipore, France). All other chemicals and reagents were of analytical grade.

### 2.2. Preparation of CR Extracts and Sample Quality Control

The rhizoma of *Cyperus rotundus* L. was purchased from Beijing Tongren Tang Pharmaceutical Co. Ltd. (Beijing, China) and was authenticated by Associate Professor Yulin Yang of the Institute of Medicinal Plant Development (IMPLAD), Chinese Academy of Medical Sciences and Peking Union Medical College (CAMS & PUMC), China. Voucher specimens (NO. 20150901) for these herbs were deposited in the National Compound Library of Traditional Chinese Medicines, China. The preparation process was as follows: 300 g of CR was crushed into small pieces and soaked in 95% ethanol by *V*:*V* (1:10) for 24 h. The same procedure was repeated twice. The two filtrates were combined and concentrated under vacuum to give CR-95E extract (11.63 g, yield 4.50%). The residues were then refluxed by 50% ethanol *V*:*V* (1:10) for 2 h. The same procedure was repeated twice. The two filtrates were combined and concentrated under vacuum to give CR-50E extract (42.09 g, yield 14.0%). The residues were then refluxed by water *V*:*V* (1:10) for 2 h. The same procedure was repeated twice. The filtrates were combined and concentrated under vacuum to give CR-W extract (19.97 g, yield 6.70%).

The accurately weighed CR-50E extract (0.5 g) was dissolved in 25 mL of methanol (*v*/*v*) and centrifuged at 13,000 rpm for 20 min. Then, 2 μL was injected for UPLC-Q-TOF/MS analysis after filtration through a 0.22 μm membrane filter. Sample analysis was carried out with a Waters UPLC instrument (Waters Corporation, Milford, MA, USA) equipped with a Phenomenex Kinetex C18 column (100 mm × 2.1 mm, 1.7 μm) at a column temperature of 45 °C, flow rate of 0.4 mL/min. The mobile phase was composed of 0.1% formic acid water (A) and acetonitrile (B). The line gradient program was carried out as follows: 5–40% B at 0–15 min; 40–100% B at 15–30 min; and 5% B at 30–32 min. The fingerprints of CR-95E, CR-50E, and CR-W are attached in the Supplementary Information (Figure S1). Nootkatone and *α*-cyperone appeared at 12.40 min and 13.60 min, respectively.

### 2.3. Cell Culture

PC12 cells were obtained from the American Type Culture Collection (Rockville, MD, USA). The cells obtained from ATCC were considered as passage 1 and the subsequently cultured cells as passage 2 and onward. The procedure of PC12 cells culture used was according to the literature [22] with some modification. PC12 cells were maintained in DMEM medium supplemented with penicillin (100 U/mL), streptomycin (100 μg/mL), 5% fetal bovine serum, and 10% horse serum at 37 °C in a humidified atmosphere of 95% air and 5% CO_2_ for 48–72 h. For all experiments, cells in the exponential phase of growth with approximately 80% confluence were used. 

### 2.4. Corticosterone Damage and Drug Treatments

All experiments are conducted in undifferentiated PC12 cells (passage 4–10).

Corticosterone was weighed and dissolved in DMEM (with 0.05% DMSO, *v*/*v*). The appropriate damage concentration and time of corticosterone were selected. In brief, different concentrations of corticosterone (10, 20, 40, 100, 200, and 400 μmol/L) were incubated with PC12 cells for different times, 24 h or 48 h, and the cell viability was determined by MTT.

To study the neuroprotective effect of different extracts from CR on PC12 cells, the PC12 cells were divided into five groups: nontreated control (control group); corticosterone (corticosterone group); CR-95E group: corticosterone plus various doses of CR-95E (0.0375, 0.375, 0.750, and 7.50 mg/mL, equivalent to the amount of raw drug calculation); CR-50E group: corticosterone plus various doses of CR-50E (0.0375, 0.375, 0.750, and 7.50 mg/mL, equivalent to the amount of raw drug calculation); CR-W group: corticosterone plus various doses of CR-W (0.0375, 0.375, 0.750, and 7.50 mg/mL, equivalent to the amount of raw drug calculation). PC12 cells were seeded at a density of 1 × 10^4^ cells/well on 96-well microplates for 24 h. After the media was removed, the cells were cultured in serum-free medium and coincubated with corticosterone and different concentrations of CR-50E using optimized damage condition.

To study the neuroprotective effect of CR-50E on PC12 cells, the PC12 cells were divided into eight equal groups: control group, corticosterone group, corticosterone plus various doses of positive medicine, corticosterone plus various doses of CR-50E (50, 125, 250, 500, and 1000 μg/mL) (CR-50E group).

### 2.5. Cell Viability Assay

All experiments were conducted in undifferentiated PC12 cells (passage 4–10).

Cell viability was evaluated using the MTT assay. Briefly, after incubation, the media was carefully removed. Then, MTT solution (final concentration, 0.5 mg/mL) was added and further incubated for 4 h at 37 °C. Subsequently, the dark blue formazan crystals formed in intact cells were dissolved with DMSO. After shaking at room temperature for 10 min, absorbance was measured at 570 nm using a microplate reader (*Bio-Rad* 550, Hercules, CA, USA). Cell viability was expressed as a percentage of the nontreated control.

### 2.6. Lactate Dehydrogenase Assay

All experiments were conducted in undifferentiated PC12 cells (passage 4–10).

PC12 cells were seeded at a density of 2 × 10^5^ cells/well in 24-well microplates for 24 h. After incubation, cells were coincubated with 200 μM of corticosterone and different concentrations of CR (125, 250, 500 μg/mL) for another 24 h. At the end of treatment, the cell suspension was centrifuged at 4000×*g* for 5 min at 4 °C, and then the supernatants were collected, whereas the cell pellets were lysed with cell lysis buffer containing 1% TritionX-100. LDH assays in supernatant aliquots and lysates were performed by using the LDH assay kit according to the manufacturer’s protocol. The wavelength to measure absorbance was 440 nm, and release rate of LDH was calculated using the formula: (Supernatant value*blank value)/[(Supernatant value*blank value) + (lysates value*blank value)]*100%.

### 2.7. Cell Metabonomics Study

All experiments were conducted in undifferentiated PC12 cells (passage 4–10).

#### 2.7.1. Cell Quenching and Extraction

For metabolite measurements, the procedure used was according to the literature [23] with some modification as follows: PC12 cells were seeded at a density of 1 × 10^6^ cells/well in six-well microplates (Costar, USA) to approximately 80% confluence. After the culture medium was removed, cells were rapidly washed with PBS. Cells were then quenched using 1.0 mL (−20 °C) methanol–water (4:1, *v*/*v*) and liquid nitrogen. Next, cells were quickly detached from the microplates using a cell lifter (Coning 3008, USA) on ice. The cell suspension was pipetted into a 2 mL centrifuge tube. Then, cells of microplates were washed with another 0.3 mL (−20 °C) methanol–water (4:1, *v*/*v*) and transferred to above 2 mL centrifuge tube. The tube was centrifuged at 1000 rpm for 5 min at 4 °C and the supernatant was transferred to a new tube.

After that, 1 mL extract solution was added to the cell debris, which was then frozen in fresh liquid nitrogen and thawed at 37 °C for 10 min, twice. That is to say, the tube was subjected to two cycles of freeze–thaw, and then vortexed twice, followed by 30 s of sonication on ice. The tube was centrifuged at 13,000 rpm for 15 min at 4 °C and the supernatant was transferred to a new tube. The cell pellet was re-extracted with 0.5 mL extract solution and vortexed vigorously, and centrifuged as well. The combined cell suspensions were dried under nitrogen stream at room temperature and frozen at −80 °C until analysis.

#### 2.7.2. UPLC-Q-TOF/MS Analysis

All cell samples were thawed at room temperature, and then the residuals of the cell samples were reconstituted and centrifuged at 13,000 rpm for 15 min at 4 °C. The supernatant was filtrated through a 0.22 μM membrane filter before LC-MS analysis.

The cell samples were analyzed on a Waters Acquity^TM^ Ultra Performance LC system (Waters Corporation, Milford, MA, USA) equipped with a binary solvent delivery system. The separation was achieved on a BEH C18 column (2.1 mm × 100 mm, i.d 1.8 μm, Waters, Milford, USA). The mobile phase consisted of 0.1% formic acid–water (A) and 0.1% formic acid–acetonitrile (B). The flow rate was kept at 0.45 mL/min during a 12 min run with the optimized gradient elution condition as follows: 1–10% B for 0–2 min, 10–70% B from 2 to 5 min, 70–90% B from 5 to 12 min, 100% B maintained for 12 min. The column temperature was 35 °C. All the samples were kept at 4 °C during the analysis.

Mass spectrometry conditions: QTOF SYNAPT HDMS system (Waters Corporation, Milford, MA, USA) was applied to collect the mass spectrometric data. The parameters were the following: capillary voltage: 3.0 kV for positive mode and 2.5 kV for negative mode; cone voltage: 20 V; extraction cone voltage: 6 V; collision energy of channel 1: 6 eV; collision energy of channel 2: 10–40 eV; scan time: 0.1 s; interscan delay time: 0.02 s; acquisition mode: centroid; *m/z* 50 to 1200; lock mass: leucine-enkephalin; lock spray frequency: 20 s; flow rate of lock mass: 80 mL/min.

In the experiment, 1989 features with high response were detected from PC12 cell samples in positive ion mode of UPLC-Q-TOF/MS data, and 231 features with low response in negative ion mode. Thus, only positive ion mode of the UPLC-Q-TOF/MS technique was used in subsequent metabonomics experiments.

#### 2.7.3. Multivariate Statistical Analysis

The raw mass spectral data were firstly recorded using the MarkerLynx Applications Manager package of MassLynx software version 4.1 (Waters Corp., Manchester, UK). Preprocess program was used to extract characteristic variables such as deconvoluted, peak identification, filtering noise, and so on. The filter parameters were set as follows: restrict retention time: 0–15 min; restrict mass: 50–1200 Da; XIC window: 0.02 min; mass window: 0.02 Da; automatically calculate peak width and peak–peak baseline noise; use the raw data during the deconvolution procedure; marker intensity threshold (count): 200; retention time windows: 0.2 min; use noise elimination; retain the isotopic peaks. The raw data were transformed into a three-dimensional data matrix including aligned ion peak areas, matched mass-to-charge ratios (*m*/*z*), and retention times. The preprocessed data were then subjected to multivariate statistical analysis.

The acquired data were introduced to SIMCA-P software package (V14.0, Umetric, Umea, Sweden). Imported data were mean-centered and pareto-scaled prior to multivariate analysis. Principal component analysis (PCA) was performed to discern the natural separation between different stages of samples by visual inspection of score plots, and to evaluate the interpretative ability and predictive ability of the established model. The data were filtered of noise by orthogonal signal correction (OSC) to remove variations from noncorrelated factors. In the orthogonal partial least squares discriminate analysis (OPLS-DA) model, samples from different groups were classified, and the results were visualized in the form of a score plot to show the group clusters, and *S*-plot and VIP plot to show differential variables contributing to the classification.

### 2.8. Apoptosis Detection

Apoptosis detection was quantified by staining cells with Annexin V-FITC and PI labeling [18]. Briefly, PC12 cells were seeded at a density of 8 × 10^5^ cells/well in six-well microplates for 24 h. After incubation, cells were coincubated with 200 μM of corticosterone and different concentrations of CR (125, 250, and 500 μg/mL) for another 24 h. At the end of treatment, harvested cells were washed twice with cold PBS and resuspended in 100 μL 1× binding buffer. Then, 5 μL of AnnexinV-FITC and 5 μL of PI were added to cells and incubated at room temperature in the dark for 10 min and 400 μL 1×binding buffer was added to each sample. The flow cytometric analysis was performed immediately.

### 2.9. Measurement of Intracellular Ca^2+^ Concentration

PC12 cells were seeded at a density of 5 × 10^4^ cells in a 25 cm^2^ flask for 24 h. After incubation, cells were coincubated with 200 μM of corticosterone and different concentrations of CR-50E (125, 250, and 500 μg/mL) for another 24 h. At the end of treatment, the cells were collected and incubated with the complete medium containing 5 μM of Fluo-3AM at 37 °C for 30 min. Subsequently, the cells were washed and resuspended with cold PBS containing 0.2% bovine serum albumin at 3 × 10^6^/mL. The cells were incubated at 37 °C for another 5 min just prior to measurement, and the intracellular Ca^2+^ concentration was determined by alternating excitation wavelengths between 340 and 380 nm with emission at 510 nm, using a fluorescence spectrophotometer (*F-4500*, Hitachi, Japan), and then the data were analyzed with customized software provided by *F-4500*. The ratio of fluorescence intensities was calculated after subtraction of the background fluorescence.

### 2.10. Western Blot Analysis

For protein extraction, PC12 cells were seeded at a density of 2 × 10^6^ cells in a 25 cm^2^ flask for 24 h. After incubation, cells were coincubated with 200 μM of corticosterone and different concentrations of CR (125, 250, and 500 μg/mL) for another 24 h. At the end of treatment, the medium was carefully removed and cells were scraped and washed with PBS. To prepare cell lysates, cells were suspended in an equal volume of PBS and lysis buffer [50 mM Tris-HCl (pH 7.4), 150 mM NaCl, 1.0% Nonidet P-40, 0.5% Na_3_VO_4_, 0.5 mM dithiothreitol (DTT), 0.5 mM phenylmethan sulfonyl fluoride (PMSF), 1 mM EDTA, and 10 mM NaF] and kept on ice for 30 min in order to protect protein from inactivity. The cell lysates were centrifuged at 12,000×*g* for 15 min at 4 °C and then the supernatants were collected and stored at −80 °C until use.

For Western blotting analysis, an aliquot (20 μL, containing 20 μg protein) of the supernatant was loaded onto an SDS gel, separated by electrophoresis, and transferred to an NC membrane. Polyacrylamide gel (10%) was used for all the electrophoresis. The transmembrane times for PLA2, CaMKII, caspase-3, and *β*-actin were 40 min, respectively. After the NC membrane was incubated with 3% BSA-TBST for 30 min, the membrane was incubated with primary antibodies overnight at 4 °C and incubated with either rabbit anti-PLA2 (1:2000 dilution), rabbit anti-CaMKII (1:2000 dilution), rabbit anti-caspase-3 (1:5000 dilution), or mouse anti-*β*-actin (1:20,000 dilution). Blots were then incubated with horse radish peroxidase-conjugated goat anti-rabbit Ig G (Beijing TDY Biotech CO., Ltd., Beijing, China) or horseradish peroxidase-conjugated goat anti-mouse Ig G (Beijing TDY Biotech CO., Ltd., Beijing, China) for 40 min at room temperature at a 1:20,000 dilution. To calculate the fold change, the density of the protein bands was determined using the Image Quant TL software provided by GE. The band densities were quantified from three different observations using Image J software (National Institutes of Health, Bethesda, MD, USA). All protein quantifications were adjusted for the corresponding *β*-actin level, which was not consistently changed by the different treatment conditions.

### 2.11. Statistical Analysis

The results were expressed as mean ± standard deviation (*x ± s*). Data were analyzed with one-way analysis of variance (ANOVA) using the Statistical Package for Social Science program (SPSS 16.0, SPSS, Chicago, IL, USA), and differences were considered statistically significant at *p* < 0.05.

## 3. Results

### 3.1. CR-50E Shows the Best Protection Effect for Corticosterone-Induced PC12 Cell Injury among the Fractions of CR

**Figure 1:**
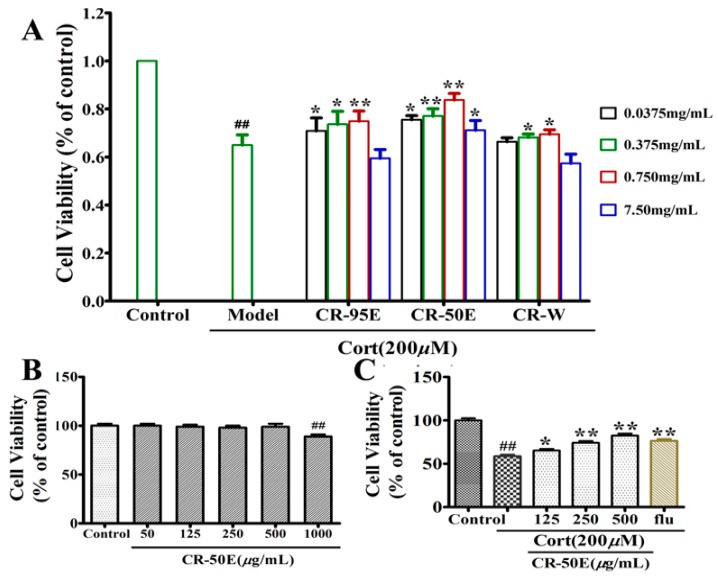
(**A**): The cell viability of different extract fractions from CR on corticosterone-treated PC12 cell; (**B**): cell cytotoxic effect was measured by the MTT assay after being cultured with various doses of CR for 24 h; **C**: cell survival was determined by the MTT assay; results are presented as means ± SD (*n* = 6). ** *p* < 0.01, compared with control group, ^##^ *p* < 0.01, compared with corticosterone (Cort).

The cytoprotective effects for corticosterone-induced PC12 cell injury among the fractions of CR (CR-95E, CR-50E, and CR-W) were evaluated by MTT assay firstly. As shown in Figure 1A, compared with the control group, corticosterone significantly reduced the cell survival rate (57.8%, ^##^ *p* < 0.01), indicating successful replication of the cell injury model. Compared with the model group, the fraction containing medium-polarity chemical constituents (CR-50E) displayed the best protection effect in spite of the 95% EtOH fraction having a weak protective effect.

### 3.2. CR-50E Increases the Survival Rate of the Corticosterone-Induced PC12 Cells

The evaluation of cytotoxic effect or proliferation effect of CR-50E on PC12 cells was carried out. The result (Figure 1B) showed that CR-50E at a concentration lower than 1000 μg/mL did not show cytotoxicity or stimulation of the proliferation of PC12 cells over 24 h. Furthermore, as shown in Figure 1C, CR-50E had a dose-dependent effect with concentration against the corticosterone-induced loss of PC12 cell viability compared with the corticosterone-treated group (125 μg/mL: 58.7% ± 3.2% of control; 250 μg/mL: 68.3% ± 4.2% of control; 500 μg/mL: 78.9% ± 2.3% of control). The above results showed that the administration of CR-50E could significantly increase the survival rate of the corticosterone-induced PC12 cells.

### 3.3. CR-50E Inhibits Cell Apoptosis Induced by Corticosterone

**Figure 2:**
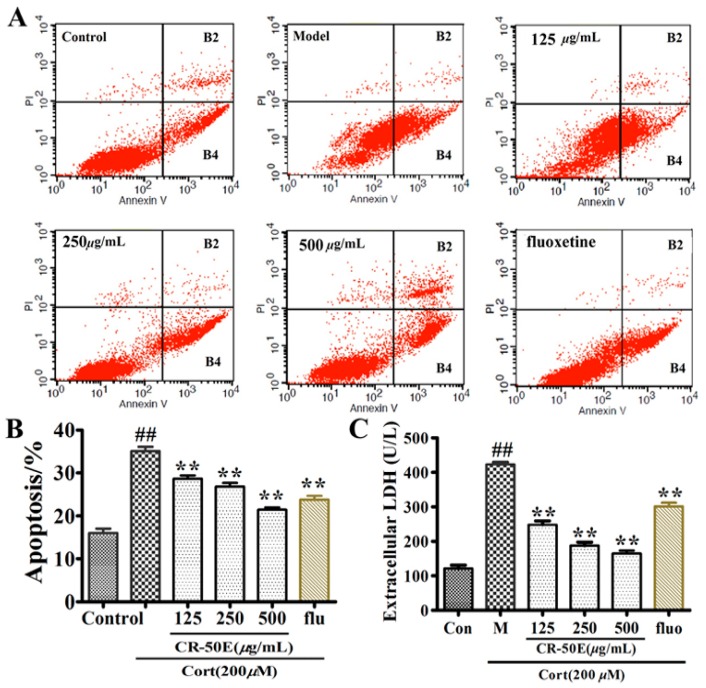
Effect of CR-50E on corticosterone-induced cell apoptosis and LDH released. (**A**)**:** The stained cells with Annexin V-FITC and PI labeling were analyzed by flow cytometry; (**B**): effect of CR-50E on corticosterone-induced distributive change of early and late apoptotic cells. (**C**): level of LDH. Results are presented as means ± SD (*n* = 6). ** *p* < 0.01, compared with control group, ^##^ *p* < 0.01, compared with corticosterone (Cort).

Cell apoptosis and LDH released assays were applied to evaluate the effect of CR-50E against the apoptosis of corticosterone-induced PC12 cells. As shown in Figure 2, exposure to corticosterone resulted in the percentage of both early apoptotic cells (AnnexinV-FITC+/PI-) and late apoptotic cells (AnnexinV-FITC+/PI+) increasing to 34.98% as compared to the normal control (20.94%), and an obvious increase of LDH released into the culture medium (423.40% ± 6.21% of control, *p* < 0.01). After the CR-50E treatment, the percentage of both early apoptotic cells and late apoptotic cells obviously decreased to 27.24% of 125 μg/mL, 24.05% of 250 μg/mL, and 18.96% of 500 μg/mL (Figure 2B). At the same time, the LDH released in corticosterone-treated PC12 cells was significantly decreased (125 μg/mL: 248.10% ± 11.21% of control; 250 μg/mL: 187.60% ± 10.21% of control; 500 μg/mL: 165.2% ± 8.35% of control) after CR-50E treatment (Figure 2C). The results showed that CR-50E could significantly inhibit the cell apoptosis induced by corticosterone.

### 3.4. CR-50E Reduces Oxidative Stress Induced by Corticosterone

Oxidative stress is widely recognized as one of the leading causes of damage and apoptosis in cells. The activities of antioxidant enzymes (SOD, CAT, and MDA) are considerable biological markers to evaluate the severity and prognosis of oxidative damage [23]. As shown in Figure S2, PC12 cells exposed to corticosterone caused decreased levels of SOD and CAT, and elevated levels of MDA compared with control. CR-50E treatment could significantly elevate the SOD and CAT activity and lower the content of MDA, which suggested that CR-50E had the effect of antioxidative damage.

### 3.5. CR-50E Improves Intracellular Abnormal Sphingolipids Metabolism Induced by Corticosterone

**Figure 3:**
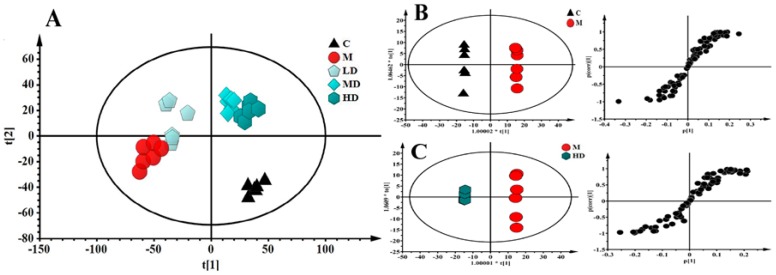
Multivariate analysis based on the UPLC-MS profiling data in positive ion mode (*n* = 6). (**A**): PCA analysis of all groups. Score plot of PCA (*R*^2^*X* = 0.665, *Q*^2^(cum) = 0.570; (**B**): OPLS-DA of control and corticosterone-treated group. Score plot of OPLS-DA (*R*^2^*X* = 0.964, *R*^2^Y = 0.904, *Q*^2^(cum) = 0.854); (**C**): OPLS-DA of corticosterone-treated and high dose CR-50E-treated group. Score plot of OPLS-DA (*R*^2^*X* = 0.834, *R*^2^Y = 0.951, *Q*^2^(cum) = 0.731). C: control group; M: corticosterone-treated group; LD: low dose CR-50E-treated group; MD: middle dose CR-50E-treated group; HD: high dose CR-50E-treated group.

The established cell metabonomics using ultra-performance liquid chromatography coupled with mass spectrometry [24] were applied to investigate the protection of CR-50E against neurotoxicity of corticosterone-treated PC12 cells (Figure S3). As shown in Figure 3A, the metabolic profiles of CR-50E treatment groups were close to the control and differed from the corticosterone-treated group, suggesting CR-50E can return the anomalous metabolic profiles to the normal levels. Furthermore, 11 altered variables made a big contribution to differentiate the groups and were found by OPLS-DA and were further confirmed by the databases such as the Human Metabonome Database (http://www.hmdb.ca/) and LIPID MAPS-Nature Lipidomics Gateway (http://www.lipidmaps.org/) (Table 1). Combined with the VIP value of identified metabolites and metabolic pathway analysis, the abnormal sphingolipids metabolism was regarded as the most likely feature of corticosterone-induced cell injury (Table 1 and Figure S4), and it is also the main way that CR-50E works. It is reported that sphingolipids (phytosphingosine, dihydroceramide, and sphinganine) were mainly ascribed to the mediation of the apoptosis and Ca^2+^ overloading [25,26]. Thus, cell metabonomics study indicated that the Ca^2+^ overloading inhibition might be how the pathway of CR-50E works through adjusting the abnormal sphingolipids metabolism.

### 3.6. CR-50E Suppresses the Expressions of PLA2, CaMK II, and Caspase-3

**Figure 4:**
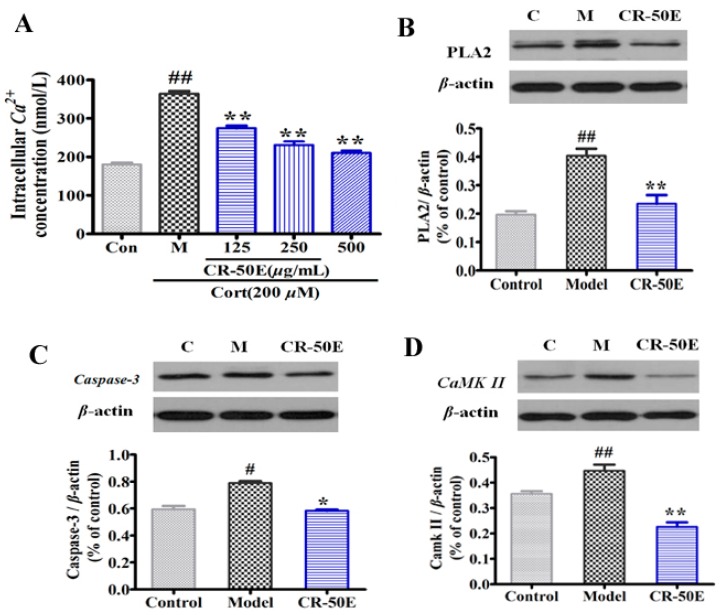
(**A**): Intracellular Ca^2+^ concentration in corticosterone-induced PC12 cells; (**B**): the protein expression level of PLA2, CaMK II, and caspase-3 in different group. Cells were exposed to 200 *μ*M of corticosterone in the absence or presence of CR-50E for 24 h. Results are presented as means ± SD (*n* = 6). ** *p* < 0.01, compared with corticosterone-treated group (Cort), ^#^ *p* < 0.05 or ^##^ *p* < 0.01, compared with control group.

Western blot analysis was used to assess the expression levels of three key proteins in calcium transport, including PLA2, CaMK II [27], and caspase-3 [28]. As shown in Figure 4A, compared with the corticosterone-treated group, CR-50E significantly decreased the fluorescence intensities of Ca^2+^ in corticosterone-treated PC12 cells detected by fluo-3AM fluorescence labeling assay. As shown in Figure 4B, typical bands of expressions of PLA2, CaMK II, and pro-caspase-3 in the model group were significantly upregulated compared with the control group (*n* = 3, *p* < 0.05 or *p* < 0.01). Pretreatment with CR-50E markedly reversed the overexpressions of these three proteins (*p* < 0.05).

## 4. Discussion

CR has exhibited pharmacological effects on neuronal diseases, such as Parkinson’s disease (PD), anxiety, and depression, and the neuroprotective effect of CR might play a vital role in exerting its effect. However, it is unclear which constituents and uncertain mechanisms are responsible for those effects of CR. The aim of the present study was to screen the active fraction of CR and evaluate its neuroprotective effect on corticosterone-induced PC12 cells. An UPLC-Q-TOF/MS-based cellular metabonomics approach was applied to investigate the potential possible molecule mechanisms of CR-50E. The present study was the first time to provide the evidence of the active fraction and potential therapeutic mechanisms of CR-50E against corticosterone-induced apoptosis in PC12 cells in vitro.

MTT assay indicated that the fraction containing medium-polarity chemical constituents (CR-50E) displayed the best protection effect in spite of the 95% EtOH fraction having a weak protective effect. Meanwhile, CR-50E significantly decreased the LDH release in a dose-dependent manner and inhibited cell apoptosis induced by corticosterone. In addition, we found CR-50E can effectively ameliorate the indicators of oxidative stress, such as SOD, CAT, and MDA. All the observations suggested that CR-50E could increase the cell viability and reduce cell apoptosis through inhibiting oxidative stress and decreasing the LDH release of PC12 cells induced by corticosterone.

**Figure 5:**
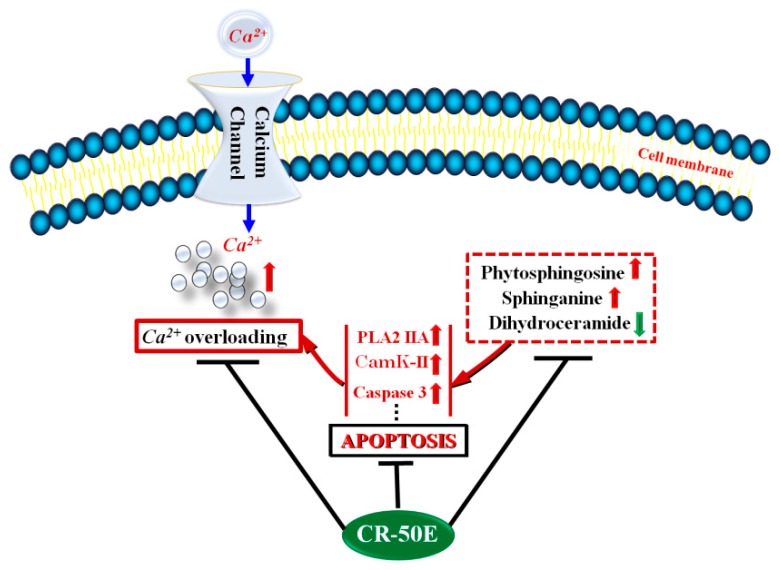
The schematic representation of the neuroprotective effect of CR-50E.

To further explore the protective effect of CR-50E on the disturbed metabolic pathways caused by corticosterone, a cell metabonomics approach was carried out. The metabolic profiles of the CR-50E-treated group were close to the control group and differed from the corticosterone-treated group, suggesting CR-50E returned the metabolic profile of the treatment group to the normal state. Eleven altered metabolites involved in sphingolipid and glycerophospholipid metabolism can be defined as potential biomarkers related to the lesions induced by corticosterone in PC12 cells. Combined with the VIP value of identified metabolites and metabolic pathway analysis, the abnormal sphingolipids metabolism was regarded as the most likely feature of corticosterone-induced cell injury. The deviations induced by corticosterone were significantly improved after treatment with CR-50E. The results showed that the neuroprotective effect of CR-50E was likely attributable to the amelioration of the sphingolipids metabolic disorders induced by corticosterone (Figure 5).

Sphingolipids (SPLs) are a class of lipid molecules that all contain a long chain base called a sphingoid backbone and are synthesized in the endoplasmic reticulum (ER). SPLs provide structural integrity to cell membranes and regulate various important cellular processes such as apoptosis, proliferation, and so on [29]. Ceramide is the center of sphingolipid biosynthesis, and is produced by hydrolysis of neurolipids [30]. Ceramide synthases catalyze the N-acylation of sphinganine (CE4) to dihydroceramide (CE3). Meanwhile, sphinganine C4-monooxygenase catalyzes the oxidation of sphinganine (CE4) to form phytosphingosine (CE2). Among the above three metabolites which changed significantly in our study, phytosphingosine (CE2) is well known to activate apoptosis [31]. It effectively promotes the opening of the mPTP, releasing cytochrome C and active caspase-3, both of which lead to cell apoptosis [32]. In our study, the accumulation of phytosphingosine (CE2) and sphinganine (CE4) and the decrease of dihydroceramide (CE3) were all observed in the corticosterone-induced PC12 cells. CR-50E significantly decreased the level of phytosphingosine (CE2) and sphinganine (CE4) and accumulated the contents of dihydroceramide (CE3), indicating that the protective effects of CR-50E might be exerted by inhibiting cell apoptosis which promotes cell survival by reduction of the cell metabolic and proliferation rate (Figure 5).

It is reported that sphingolipids act as secretagogues that trigger an exocytotic response, either by mobilizing Ca^2+^ from internal stores or by increasing extracellular Ca^2+^ influx via activation of voltage and store-operated calcium channels [25,26]. Thus, to further validate the participation of Ca^2+^ overloading and the related proteins in the process of corticosterone-induced injury, three proteins (PLA2 IIA, CaMK II, and cleaved caspase-3) were chosen to verify. The levels of the above three proteins were significantly upregulated in the corticosterone-treated PC12 cells. Pretreatment with CR-50E markedly suppressed the corticosterone-induced overexpression of PLA2, CaMK II, and cleaved caspase-3, which confirms that the cellular protecting mechanism of CR-50E on PC12 cells from corticosterone-induced neurotoxicity might work through antagonizing the overload of Ca^2+^ and inhibiting neuronal apoptosis (Figure 5).

Moreover, it is reported that the accumulation of dihydroceramide (CE3) stimulates autophagy, possibly together with other transcriptional activities directed by ER sensors, promoting the survival by reduction of cell metabolic and proliferation rate [33]. Endoplasmic reticulum stress (ERS) is a self-protecting regulation system which is involved in various physiological and pathological conditions [33]. Our study found the contents of dihydroceramide (CE3) was accumulated after CR-50E treatment, indicating that CR-50E might activate ERS and eventually reduce the cell apoptosis. This result needs further experiments to research and verify.

In addition, the existing empirical studies are inconsistent in the active fraction of CR. Research groups have demonstrated that the 95% ethanol extract [11] and the ethyl acetate and n-butanol fractions from the 70% ethanol extract of CR [10] demonstrated antidepressant effects. Additionally, it has been reported that the volatile oil site of CR improved the anxiety behaviors of mice exposed to chronic restraint stress [12]. In this study, the active site was 50% ethanol fraction which was extracted after 95% ethanol, and the chromatogram showed that the polarity of chemical compositions of CR-50E extract was greater than CR-50E (Figure S1). Compared with the reference substance, the content of *α*-cyperone (the main active ingredient of CR reported at present) in CR-50E extract is relatively low. All the results indicated that the medium- or high-polarity components may be the main active constituents. The chemical composition analysis and identification of the obtained active fraction will be carried out in the follow-up research.

## 5. Conclusions

The present study firstly suggests that CR-50E exerts a neuroprotective effect on corticosterone-induced neurotoxicity in PC12 cells through cell metabolomics and molecular biological method. CR-50E plays the role of nerve cell protection via the regulation of cell sphingolipids metabolism, inhibition of cell apoptosis though attenuation of the intracellular Ca^2+^ overloading, and activation of ERS. This neuroprotective effect may be one of the acting mechanisms that account for the in vivo antidepressant activity of CR-50E. Our findings will help to investigate the pharmacodynamic active constituents of CR and provide a basis for its clinical application.

## Figures and Tables

**Table 1:** The variation tendencies of the 11 altered variables related to the lesion in corticosterone treated PC12 cells after *CR-50E treatment*.

NO.	Rt(min)	*m*/*z*	Metabolite	VIP	M/C ^a^	CR-50E/M ^b^
CE1	5.32	274.2749	Unidentified	2.01	↓ **	↑ **
CE2	5.35	318.3011	phytosphingosine	3.93	↑ **	↓ **
CE3	5.37	362.3268	dihydroceramide	2.24	↓ **	↑ **
CE4	5.91	302.3058	Sphinganine	2.21	↑ **	↓ **
CE5	6.20	265.1473	5*β*-cyprinol sulfate	3.59	↓ **	↑ **
CE6	6.78	309.1732	Unidentidied	2.32	↓ **	↑ **
CE7	7.13	478.2930	LysoPE(18:2(9Z,12Z)/0:0)	1.05	↓ **	↑ **
CE8	7.16	506.3243	LysoPE(0:0/20:2(11Z,14Z))	1.29	↓ **	↑ **
CE9	7.64	653.3003	DG (36:5)	1.41	↓ **	↑ *
CE10	7.99	480.3085	LysoPE(0:0/18:1(11Z))	1.09	↑ **	↓ **
CE11	8.02	508.3398	LysoPC(P-18:0)	1.22	↑ **	↓ **

^a^ Compare to control group; ^b^ Compare to corticosterone treated group; C: control group, M: corticosterone-treated group, *CR*: *CR-50E* group; Up-regulated (↑) and down-regulated (↓) (*p < 0.05, ** *p* < 0.01, *** *p* < 0.001) LysoPE: lysophosphatidylethanolamine; LysoPC: Lysophosphatidylcholine.

## Supplementary Materials

The following are available online at https://www.mdpi.com/2218-1989/9/11/244/s1, Figure S1: UPLC chromatograms of the *CR-95E, CR-50E and CR-W* extracts, Figure S2: Effect of *CR-50E* on corticosterone-induced antioxidants enzymes (SOD, CAT, and MDA) activity; Cells were exposed to 200 *μ*M of corticosterone in the absence or presence of *CR-50E* for 24 h. Results are presented as means ± SD (*n* = 6). ** *p* < 0.01, compared with corticosterone-treated group (Cort), ^#^ *p* < 0.05 or ^##^ *p* < 0.01, compared with control group, Figure S3: Base peak intensity (BPI) chromatograms of UPLC-Q-TOF/MS in positive ion mode from the PC12 cell samples in each group. A: control group; B: corticosterone-treated group; C: High dose CR-50E-treated group, Figure S4: Summary of metabolic pathway analysis with MetaboAnalyst 3.0. Each point represents one metabolic pathway; the size of dot and shades of color is in positive correlation with the impaction of the metabolic pathway, Table S1: Summary of metabolic pathway analysis with MetaboAnalyst 3.0. based on the identified metabolites.
