# Supplementary material for: Neuroprotective Effect of Cyperi rhizome against Corticosterone-Induced PC12 Cell Injury via Suppression of Ca2+ Overloading
Source: Metabolites. 2019 Oct 23;9(11):244. doi: 10.3390/metabo9110244 (PMC6918173; doi:10.3390/metabo9110244)
Supplement: Supplementary file 1 [file metabolites-09-00244-s001.pdf]

Electronic Supplementary Information

Neuroprotective effect of *Cyperi rhizome* against corticosterone-induced PC12 cells via suppress Ca^{2+} overloading

Hongmei Jia, Yang Liu, Meng Yu, Hai Shang, Hongwu Zhang, Liyan Ma, Tao Zhang, Zhongmei Zou*

Institute of Medicinal Plant Development, Chinese Academy of Medical Sciences and Peking Union Medical College, No. 151 Malianwa North Road, Haidian District, Beijing 100193, China

Tabe S**1** Summary of metabolic pathway analysis with MetaboAnalyst 3.0.based on the identified metabolites

Metabolic pathway	Total	Expected	Hits	Raw p	Impact
Sphingolipid metabolism	21	0.074893	2	0.0020808	0.14286
Glycerophospholipid metabolism	30	0.10699	1	0.10265	0.04444
Steroidhormone biosynthesis	70	0.24964	1	0.22622	0.01699

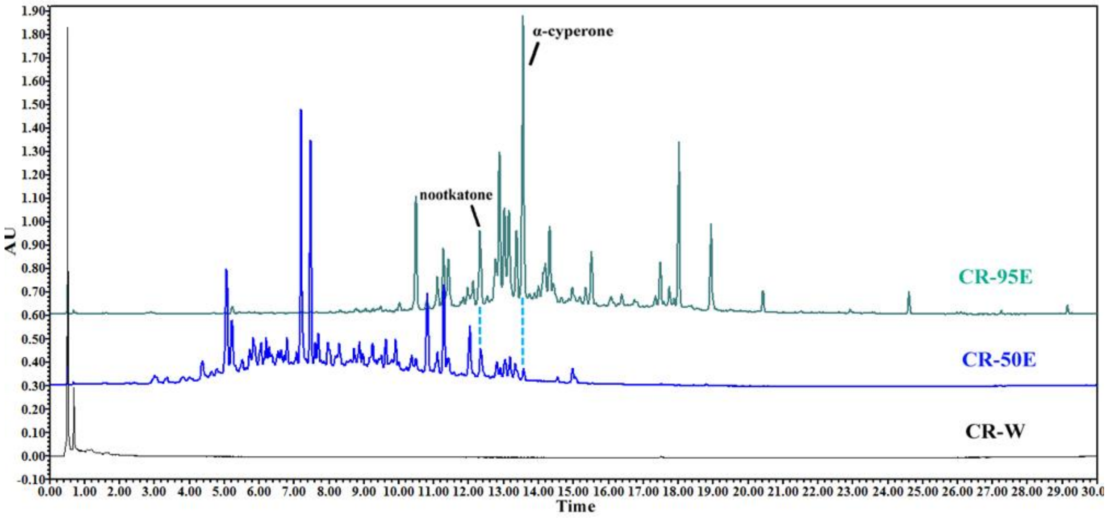

Fig. S1 UPLC chromatograms of the *CR-95E, CR-50E and CR-W* extracts.

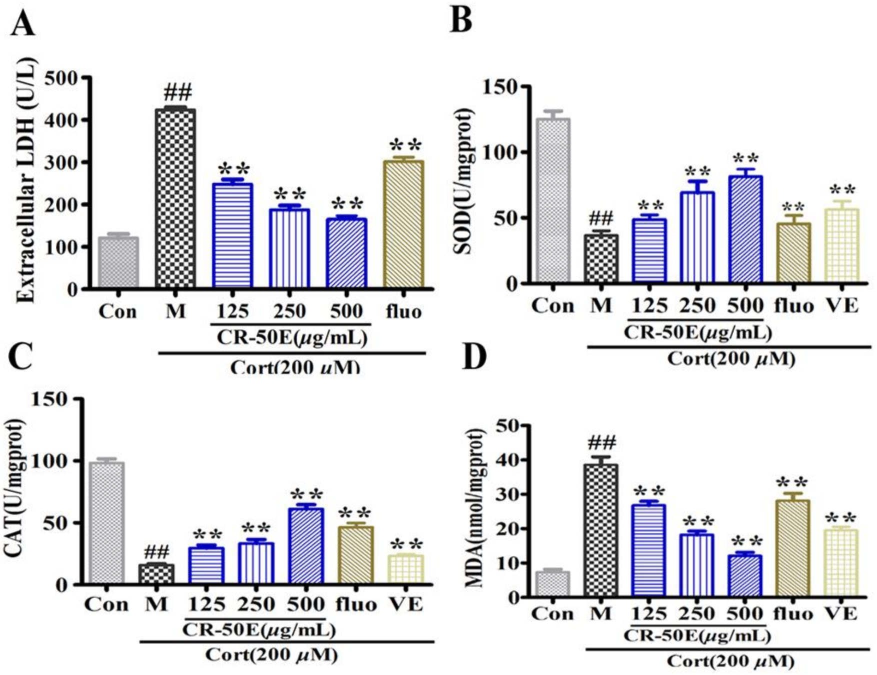

Fig S2. Effect of *CR-50E* on corticosterone-induced antioxidants enzymes (SOD, CAT, and MDA) activity; Cells were exposed to 200 μM of corticosterone in the absence or presence of *CR-50E* for 24h. Results are presented as means ± SD (n=6). ***$p < 0.01$, compared with corticosterone-treated group (Cort), $^{\#}p < 0.05$ or $^{\#\#}p < 0.01$, compared with control group.

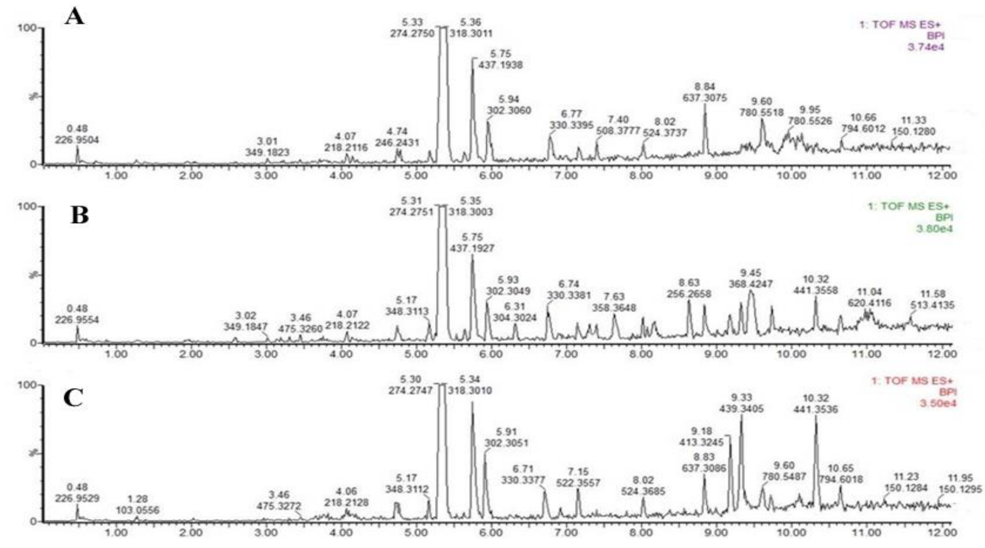

Fig. S3 Base peak intensity (BPI) chromatograms of UPLC-Q-TOF/MS in positive ion mode from the PC12 cell samples in each group. A: control group; B: corticosterone-treated group; C: High dose CR-50E-treated group.

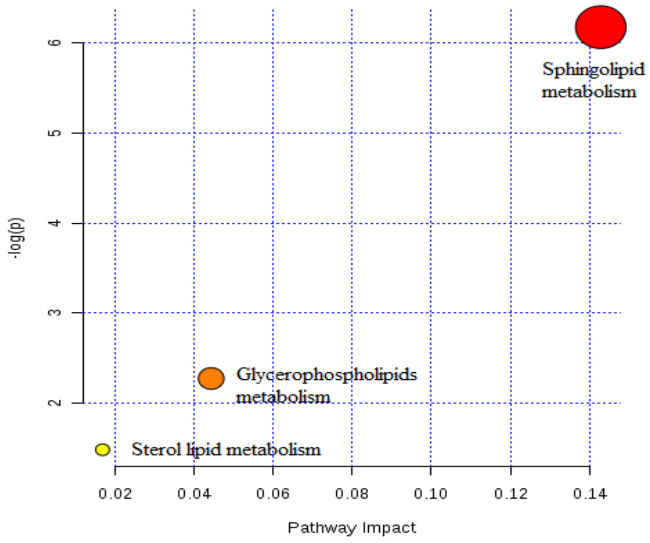

Fig. S4 Summary of metabolic pathway analysis with MetaboAnalyst 3.0. Each point represents one metabolic pathway; the size of dot and shades of color is in positive correlation with the impaction of the metabolic pathway.
